# A Comprehensive Understanding of Psychomotor Agitation: From Causes to Hospital Care

**DOI:** 10.2174/011570159X340145250109080932

**Published:** 2025-04-22

**Authors:** Antonio Del Casale, Jan Francesco Arena, Christian Napoli, Fabiano Grassi, Serena Mancino, Cristina Di Legge, Barbara Adriani, Giovanna Parmigiani, Alessandro Vento, Giorgio Veneziani, Carlo Lai, Stefano Ferracuti

**Affiliations:** 1 Department of Dynamic and Clinical Psychology and Health Studies, Faculty of Medicine and Psychology, Sapienza University of Rome, Rome, 00185, Italy;; 2 Department Emergency and Admissions, Unit of Psychiatry, ‘Sant'Andrea’ University Hospital, Rome, 00189, Italy;; 3 Department of Medical-Surgical Sciences and Translational Medicine, Faculty of Medicine and Psychology, Sapienza University of Rome, Rome, 00185, Italy;; 4 National Institute for the Promotion of Migrant Population Health and the Fight Against Poverty Diseases, Via di S. Gallicano 25/a, Rome, 00153, Italy;; 5 Unit of Risk Management, ‘Sant'Andrea’ University Hospital, Rome, 00189, Italy;; 6 Department of Neuroscience, Mental Health and Sensory Organs (NESMOS), Faculty of Medicine and Psychology, Sapienza University, Rome, 00189, Italy;; 7 Department of Human Neuroscience, Faculty of Medicine and Dentistry, Sapienza University of Rome, Rome, 00185 Italy;; 8 Mental Health Centre, Department of Mental Health, Local Health Authority Rome 2, via Monza 2, Rome, 00182, Italy;; 9 Observatory on Dependencies and Sub-threshold Psychiatric Disorders, Rome, Italy

**Keywords:** Emergence agitation, psychiatry, evidence-based emergency medicine, pediatrics, delirium, psychopharmacology, involuntary treatment, clinical decision-making

## Abstract

Psychomotor agitation is a syndrome characterized by abnormal psychic and motor activation, with a concrete possibility of escalation to aggression or violence. It represents a frequent reason for attending emergency departments and can stem from various organic conditions and mental disorders. In emergency settings, prompt identification of the possible underlying cause and optimal management are crucial to ensure the safety of both patients and healthcare providers. However, current management strategies are based on evidence focused on too specific clinical contexts or aspects of patient care. This narrative review aims to consolidate available evidence on clinical-diagnostic evaluation, severity assessment, patient-specific interventions, and hospital management. Patients should always be approached using de-escalation techniques while providing a rapid and systematic assessment of the key differential elements between organic and psychiatric causes. Pharmacological interventions are recommended as secondary measures to ensure safety and should be directed at facilitating the therapeutic relationship. Physical restraints and seclusion should be used only as a last resort, for the shortest duration, and under strict medical supervision. There is a pressing need for the systematic organization of evidence into effective guidelines to optimize the clinical approach to psychomotor agitation, improving both patient outcomes and safety in emergency settings.

## 1 INTRODUCTION

Psychomotor agitation (PMA) is a multifactorial syndrome characterised by increased and overpowering motor activity, irritability, and hyperresponsiveness to internal/external stimuli [1]. Considering PMA as a clinical dimension, affected patients could experience a state of emotional arousal and possible cognitive alterations that imply a concrete risk of escalation to aggression and violence [2]. Discriminating between the various aetiologies of PMA can present a diagnostic challenge, underscoring the critical significance of an accurate diagnostic assessment for effective PMA management [3]. In clinical practice, three degrees of PMA (mild, moderate, and severe) can be identified based on the patient’s behaviour [4]; however, the assessment strategies have not yet been standardized. This is relevant if it is considered that possible interventions range from verbal de-escalation to physical restraint or seclusion and are usually carried out in emergency settings [5].

PMA represents one of the main reasons for attending primary emergency settings in patients with a mental disorder and is present in around 4.6% of emergency psychiatric interventions, according to “The STAGE study” [6]. Moreover, PMA often recognizes organic causes, which may be associated with delirium or behavioural disorders caused by intoxication, infectious conditions, metabolic disorders, physical trauma, and other medical conditions [7].

In psychiatry, PMA, as a transnosological phenomenon, can regard a broad number of different diagnostic categories and clinical dimensions [8]. Psychiatric emergencies are defined as severe behavioural changes and encompass conditions such as PMA. Even though PMA is not always associated with violent behaviour [9], in some cases, PMA, hostility, aggressiveness, and violence are perceived as if they were a single category rather than a clinical dimension, which potentially leads to forms of stigma towards affected patients, impacting negatively on the clinical management of PMA. Defining adequate approaches may help reduce the stigma associated with these conditions [9] and optimize the clinical management of PMA. Moreover, in psychiatry wards, agitation episodes increase the re-admission rate with a longer length of stay, drug consumption, and higher healthcare costs [10].

Although agitation etymologically refers to a specific form of motion, the adjective “psychomotor” highlights movement resulting from mental activity and underscores the importance of accurate differential diagnosis, assessment, and definition of PMA [11]. Furthermore, the absence of a universally accepted definition and the limited availability of direct epidemiological investigations impede our understanding of this condition [6].

At present, many valuable studies and expert consensus have addressed the problem of PMA management. However, given the magnitude of the topic, they are inevitably focused on specific populations [1, 2, 4, 12], aspects [13], or interventions [14]. To the best of our knowledge, there is a lack of literature focusing on the complexity of the overall management of PMA, considering its aetiology, de-escalation techniques, pharmacological interventions, and the challenges and management in a hospital setting.

The aim of this narrative review is to conduct a critical analysis of the main literature topics on PMA and present a comprehensive summary of its various facets. This includes an exploration of the initial approach to patients exhibiting PMA, as well as an examination of clinical management strategies, encompassing assessment, treatment modalities, available guidelines, and the intricacies of hospital management. Our objective is to offer a thorough overview of the issue, with the intention of raising further inquiries that may contribute to the development of more efficient and personalized management and treatment approaches for PMA.

## 2 AETIOLOGY

When dealing with an agitated individual who exhibits anger or a lack of control, verbal de-escalation techniques are adequate in the first place, even in aggressive patients [7, 15]. Understanding the underlying causes of agitation is essential to identify the most appropriate management. This section explores the etiological factors associated with agitation, focusing on organic and psychiatric causes. When PMA occurs in patients without relevant anamnestic information or provisional diagnosis, this condition should be presumed to derive from general medical cause until proven otherwise [1], particularly in over-45-year-old patients [7]. Therefore, it is recommended to provide routine medical examinations including complete vital signs evaluation, clinical examination, blood glucose measurement, and toxicological urine tests at least [1].

The clinical assessment directs the most appropriate diagnostic pathway. First, it’s important to assess the presence of delirium, a global disturbance of consciousness with various possible underlying organic causes, that in one-third of cases present agitation (hyperactive or excited delirium) [7]. Delirium can be pictured within three subtypes, depending on the psychomotor activity: hyperactive, hypoactive and a mixed form. It usually shares with PMA an acute onset, and marked deficits in consciousness, inattention, disorganization, and visuospatial and perceptive disturbances characterize it. An important clinical element is that delirium often shows fluctuations in behaviour and symptomatology over time [16]. A history of recent surgery [17], an ongoing infectious episode, the comorbid presence of dementia [18], epilepsy [19], sleep deprivation or dehydration are to be considered among some of the elements potentially leading to the development of delirium.

In the absence of a frank traumatic event or without characteristic co-occurring symptoms, the differential diagnosis may be challenging for clinicians, especially considering the emergency setting in which the evaluation usually occurs. Organic aetiology should be considered with particular attention for cases where abnormal or fluctuating vital signs, physical examination findings, signs of alcohol/drug intoxication/withdrawal [20, 21], and poor/worsening general conditions are present. These elements should serve as clinical “markers,” requiring a careful general medical evaluation.

Agitation commonly occurs in psychiatric disorders such as psychotic disorders, mania, agitated depression, personality disorders, anxiety disorders, neurodevelopmental disorders, or as a reaction to traumatic situations [1]. In psychiatry, PMA is more commonly associated with bipolar disorder and schizophrenia spectrum disorders and shows potential for unpredictable escalation to dangerous behaviours. Substance abuse and persecutory delusions are independently associated with PMA, whose milder forms may predict violent and aggressive behaviour [22]. Some behavioural antecedents can be identified as risk factors for PMA escalation in psychiatry settings. Clinicians and staff members should pay attention to hyperactivity, impatience, loudness, hostile behaviour, intimidation, blocking escape routes during interactions, prolonged eye contact, liable mood, physical/verbal self-abusiveness, demanding and uncooperative behaviour, repetitious verbal activity, expression of anxiety and apprehensiveness as they often precede aggression in the agitated patient [23].

Nevertheless, in patients with a diagnosed mental disorder, clinicians may be prone to underscore the possibility of a medical cause underlying PMA. Some “red flags” should be considered as possible indicators of organic aetiology: over 45 age, presentation non-consistent with previous relapses, and comorbid substance use disorder [24]. Moreover, serotonin syndrome and neuroleptic malignant syndrome should always be ruled out [25]. Anyway, when PMA is related to a mental disorder, it usually presents an acute/subacute onset without frank anomalies in the level of consciousness [2] (Table **1**) [1, 7, 24, 26-34].

**Fig. (1):**
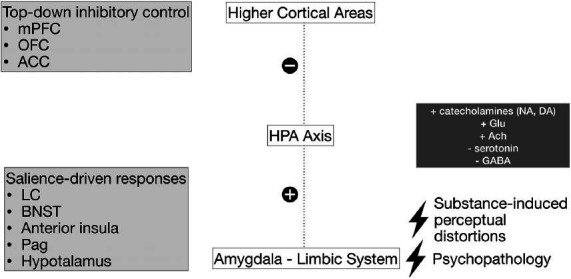
Overview of some agitation underpinning mechanisms. **Abbreviations**: ACC: anterior cingulate cortex; Ach: acetylcholine; BNST: bed nucleus of the stria terminalis; DA: dopamine; GABA: γ-Aminobutyric acid; Glu: glutamate; HPA Axis: hypothalamic-pituitary-adrenal axis; LC: locus coeruleus; mPFC: medial prefrontal cortex; NA: noradrenaline; OFC: orbitofrontal cortex Pag: periaqueductal grey matter.

PMA can have many underlying causes. However, specific pathways leading to agitation have been identified, shedding light on some neurobiological aspects. Some pivotal elements leading to psychic agitation are the increased or erroneous threat perception and the disrupted autonomic response. Fear and aggression-related pathways are deeply intertwined. Aggressive behaviour results from the interaction of the threat network, the frustrative-non reward network and the fronto-parieto-insular network, revealing the involvement of both situational and trait factors [35]. For example, in patients affected by dementia, agitation is framed within a wider behavioural dysfunction involving the frontal lobe (dorsolateral prefrontal cortex, orbitofrontal cortex, anterior cingulate cortex), locus coeruleus, anterior temporal lobe, and superior parietal cortex [36]. Thus, PMA and its escalation may result from an altered interplay between higher cortical areas, hypothalamic-pituitary-adrenal axis and limbic system, possibly exacerbated by factors such as psychopathology (*e.g*., borderline or antisocial personality disorder), substance-induced perceptive distortion or other external situations (Fig. **1**) [37].

On the one hand, in schizophrenia, PMA was associated with higher C-reactive protein, FT3, and creatinine levels and with lower TSH levels [38]; instead, in Alzheimer’s disease, a potential role of IL-1b and natural killer cell activity has been highlighted [39]. These findings suggest that a broader multisystemic approach to PMA may highlight its mechanisms in different conditions and pave the way for further research on the specific aetiology of PMA.

## 3 ASSESSMENT OF PMA

A standard clinical evaluation system of PMA in emergency settings is still lacking. Nevertheless, in schizophrenia, three degrees of severity can be identified based on the patient’s behaviour. Mild agitation would be characterized by hyperreactivity, psychomotor tension, and flashes of anger, while, moderate agitation would present verbal outbursts, uncooperativeness, fear, or paranoid behaviour. Finally, verbal/physical aggression, attention disturbances, and disorganization would distinguish a state of severe agitation [4, 5].

According to a systematic review conducted by Zeller *et al*. (2010), 13 validated scales have been developed and recommended for use across multiple clinical settings to evaluate agitation (Table **2**) [40-45]. Most of them were designed or further applied in psychiatric settings to assess aggressive-like-behaviour and the risk for violence. More recently, the Brief Agitation Rating Scale (BARS) has been recommended for the assessment of psychomotor activity, again in psychiatric settings, after a comparison with the Sedation-Agitation Scale (SAS) [41]. In psychiatric clinical and research settings, the Corrigan Agitated Behavior Scale (ABS) has found wide employment as well [42, 43]. Moreover, in several clinical trials, it is possible to find the application of the Agitation-Calmness Evaluation Scale (ACES) score, increasing the number of available tools from 13 to 14 [44, 45]. Even if some of these tools may be applied in different clinical settings, the non-systematic search for PMA-specific transdiagnostic assessment tools on databases such as PUBMED has so far proven fruitless (keywords: psychomotor agitation, assessment tool, assessment questionnaire, assessment score).

An optimal assessment tool would provide benefits, on the one hand, on the evaluation of treatment strategies according to the severity of PMA, on the other hand, on the prediction of escalation to aggressive/violent behaviour. However, it’s important to underline how the emergency setting is not suitable for the administration of questionnaires most of the time, as it may further trigger patients and complicate the staff’s work [1]. Considering practical applications, only a few scales can be administered within a few minutes (*e.g*., PANSS-EC, OAS, CGI-A, BVC, VSC), making most of them unsuitable for clinical use. An ideal tool should be easy to manage for every healthcare professional and further research is still needed in this field. Thus, a promising path may be represented by artificial intelligence (AI). According to the study protocol published by Zhang *et al*. this year, the research team will test an AI-AntiDelirium tool that could potentially assess risk factors for delirium in intensive care units and optimize treatment with a personalized AI-based approach [46]. This is an interesting approach to multifactorial medical conditions and could be valuable in PMA management, especially with the integration of artificial intelligence or informatics-based methods. These new perspectives should encourage further research. For the moment, only time will tell.

## 4 TREATMENT OF PMA

### 4.1 De-escalation Techniques

A fundamental task of PMA management is to prevent the severe consequences of an eventual escalation. The prompt recognition of PMA, as well as an appropriate assessment, would contain the patient's state, leading to a reduced risk of an escalation of aggression and violence directed towards oneself or others [1, 47]. Even if the specific combination of factors underlying aggressive behaviour is still to be determined, agitation, aggression, and violence are generally seen as part of the climax of symptoms progressively evolving one into another [48].

Several authors analysed the progression of aggressive events according to an aggression cycle characterized by the sequential succession of different phases [49-51]. Aggression and violence would arise from an initial phase, in which a triggering event has an emotional and cognitive impact on the person. This is followed by an escalation phase, characterised by an increase in agitation, until the crisis phase, in which the aggressive behaviour occurs [50]. Lastly, a gradual return to baseline and a depression phase, characterised by physical and mental exhaustion, conclude the cycle [50].

Environmental modifications and verbal de-escalation techniques (DT) should be the first-line interventions, which could be particularly relevant, considering that inadequate management of the early stages of PMA could lead to inappropriate use of coercive techniques, potentially precipitating aggression and/or violence [2, 23]. Indeed, seclusion and restraint practices have generally shown negative effects and should be used carefully as last-line interventions, underlining the crucial importance of the first phases of PMA management [52-54].

De-escalation is described as a psychosocial intervention consisting of a set of procedures designed to interrupt the possible harmful evolution of PMA, and which encompasses both verbal and non-verbal communication skills [49, 55-57]. It is possible to consider de-escalation as a dynamic procedure in which specific verbal and non-verbal cues are used to establish a therapeutic alliance [2, 15, 40] aimed to “help the patient in calming” [15]. Considering the interpersonal dimension that sustains the therapeutic alliance, in this context, the clinicians’ state itself acquires a pivotal role [15, 58]. It is noteworthy that, when approaching the agitated patient, the clinician should remain aware of her/his emotional and physical state, remaining calm to de-escalate the patient’s state [58]. Indeed, a frightened clinician would not be effective in reaching patient safety [15].

Most studies analyse de-escalation in the context of the aggression cycle [49], with specific interventions tailored based on the first two phases. At the beginning (phase 1), clinicians should principally respond with empathy and coordination of the staff, reducing the presence of threatening objects [51]. When the escalation starts (phase 2), and the patient starts to show verbal aggression, clinicians should “talk down” and actively employ DT [51].

Interestingly, some models have described de-escalation as a “process” that begins with the delimitation of the situation, proceeds with the clarification of the criticality together with the patient, and ends with the achievement of a solution [59]. More recently, Berring and colleagues (2016) within the symbolic interactionism theoretical framework, suggested that an effective de-escalation needs the ability for the clinician to imagine the patient’s perspective and, more generally, the staff's ability to “assume the role of the other” [60]. In this regard, empathic communication is related to the ability to recognise the mental state of the other person, according to the Theory of Mind [61], and some authors posed how these skills would establish an essential bridge with the other one to open the way to de-escalation [62].

Verbal de-escalation further fosters the doctor-patient relationship, enabling the latter to become an active partner in their assessment and treatment. One staff member should take the role of communicating with the patient experiencing agitation to assess the situation for safety in a designated area: a dialogue should be engaged using emotional regulation to control verbal and non-verbal expressions of anxiety/frustration while carrying out de-escalation in a non-confrontational manner [57]. Verbal de-escalation may ensure a period of relative calm, while restraint may need important staff involvement and much time [63].

Noteworthy, DT includes several non-verbal cues [64]. Egan (1975), emphasizing the non-verbal skills in therapeutic communication, proposed the importance of making sure to be physically present to a client. The author proposed “SOLER” as a model to sustain effective therapeutic communication, in which the listener should “Sit squarely,” with an “Open posture” (*e.g*., without crossing her/his legs), communicating the interest in what the person is talking about, by “Leaning towards the other,” maintaining “Eye contact,” and trying not to demonstrate nervousness - “Relax” [65]. More recently, the “SURETY” was proposed as a new model that adds the “Touch” and “Your intuition” as important components in creating a practical therapeutic space [66]. The new model emphasizes how the appropriate use of touch (*e.g*., touching the hand or the lower arm) could communicate compassion and empathy. Moreover, in this model, clinicians’ intuition acquires primary importance, and it is fundamental to sustain all other components [66].

Nevertheless, regarding psychosis-induced aggression, DT seems to be accepted as a “good clinical practice.” Still, they are not supported by evidence from randomised controlled trials [55] with the definition of a broad unsearched field in the management of PMA in hospital-related settings resulting in relevant clinical unmet needs. A similar situation regards non-psychosis-induced aggression, where the evidence-based rationale in the application of DT remains uncertain [67] with minimal evidence for the effectiveness of their application and of dedicated staff training [68]. However, despite the shortage of evidence, employing DT finds wide application and good feedback in clinical practice, relying mainly on staff expertise and available guidelines [15].

### 4.2 Pharmacological Interventions

If a de-escalation-based approach proves ineffective in restraining agitated behaviour, clinicians should consider using pharmacological interventions while still maintaining a safe environment and employing verbal and non-verbal precautions [69]. It is advisable to seek agreement with the patient before administering any treatment, regardless of the clinical presentation, and to always choose a non-traumatic/non-invasive route of administration. The main achievement of a drug in the case of PMA is to swiftly calm patients without causing excessive sedation, promoting clinical evaluation and the patient’s safety [2]. According to a consensus provided by the American Association of Emergency Psychiatry in 2012, the general pharmacological classes used in the treatment of PMA are first- and second-generation antipsychotics (SGAs) and benzodiazepines (BDZ) [13]. Generally, antipsychotics should be considered as first-line options when PMA has a psychiatric underlying cause or if the aetiology is yet to be defined [2].

### 4.3 Alcohol Intoxication and Withdrawal

When alcohol triggers a PMA episode, the choice should fall on a BDZ if the patient shows abstinence symptoms or on an antipsychotic in the case of intoxication [70]. In the former case, the risk of epileptic seizures and *delirium tremens* represents one of the major concerns when selecting therapy and BDZ is the most appropriate class [71]. In the latter case, the clinician should consider that respiratory depression could be exacerbated by BDZ administration (especially when administered parenterally), and rather consider an antipsychotic [13]. However, since in alcohol withdrawal, BDZ can still be associated with respiratory depression and paradoxically agitation, in intensive-care units for patients affected by severe forms of withdrawal phenobarbital-based treatment protocols have been recently investigated and proposed as safer and effective [72, 73]. In this specific case of alcohol withdrawal, the treatment should be represented by BDZ addressing symptoms and preventing seizures, combined with intravenous Vitamin B1 and glucose to prevent Wernicke’s encephalopathy [74]. In PMA, SGAs are usually preferred to FGAs for their lower risk of QTc prolongation and extrapyramidal symptoms. However, if PMA is caused by central nervous system depressants, such as in alcohol intoxication, the use of SGAs shows a less sedative effect [75], and the use of haloperidol is supported by the literature as the preferred FGA [13]. Notably, recently, a growing body of evidence promotes the use of SGAs such as risperidone and olanzapine as well [13, 76], paving the way for their future application in a field where haloperidol still represents the first choice [13].

### 4.4 Psychiatric Emergency Settings

When PMA is caused by psychiatric conditions, an SGA should be the preferred choice, possibly administered orally [13]. The main reasons are a lower extrapyramidal effect rate and a comparable calming effect to haloperidol, even if randomized controlled trials evaluating SGAs in this field are incredibly scarce [77]. Risperidone, olanzapine and ziprasidone are the most investigated and should be considered in the first place. On the other hand, some SGAs may not be suitable for emergencies due to their specific characteristics. Aripiprazole has a slightly reduced calming effect, quetiapine carries a relatively high risk of orthostatic hypotension, and clozapine is only approved for treatment-resistant schizophrenia, with a tolerability profile unsuitable for emergency settings. Further active principles, such as lurasidone, iloperidone and asenapine, still need further investigation in PMA treatment and should not be considered in the first instance [13].

FGAs, on their side, are widely used in the treatment of acute agitation. Haloperidol is the most extensively studied FGA and is commonly used as a reference in comparative studies. It is still commonly considered the first choice, even if a recent meta-analysis showed scarce benefits in rapid tranquilization, supporting only its use in combination with promethazine and outlining promising evidence in favour of olanzapine oral administration instead [77]. Consistently with available evidence, a further review underlines how in aggressive behaviour, haloperidol should be used only in combination with BDZs or promethazine and supports the efficacy of monotherapy with SGAs [78]. Among other FGAs, chlorpromazine (CPZ) should be avoided, when possible, due to the risk of hypotension [47], even if the available evidence is limited and outdated [79]. Haloperidol, as a butyrophenone, has less anticholinergic and epileptic threshold impact compared to phenothiazines [80], making the latter less suitable for rapid tranquilization in PMA. Interestingly, haloperidol should be used as a first-line treatment for alcohol intoxication, especially when BDZs have already been used, as discussed in the previous paragraph [13]. Moreover, loxapine, as an FGA with a peculiar high affinity to 5-HT_2A_ receptors, has shown promising potential in PMA management [81], although it appears in a relatively small number of clinical trials.

Augmentation therapy with a BDZ can be considered in agitated patients and some authors have underlined how BDZs and promethazine could have a place in PMA exhibited by patients already treated with an SGA [47].

In clinical practice, different psychoactive drugs are often administered together. The combination of haloperidol and lorazepam has been studied, highlighting a lower incidence of extrapyramidal effects than haloperidol alone [82], providing a more rapid reduction of agitation and resulting superior to the combination of haloperidol, lorazepam and diphenhydramine [83]. As for SGAs, there is still no sufficient evidence supporting their combination with BDZs or their use in alcohol intoxication [13]. According to the FDA, parenteral BDZs should not be administered with intramuscular olanzapine [1]. This warning is in contrast with some of the available evidence [84] and underlines the necessity of further investigations better to evaluate the safety profile of this treatment combination.

BDZs such as lorazepam, diazepam and clonazepam may be preferred in patients showing stimulant intoxication (*e.g*., cocaine) or alcohol withdrawal with particular attention to the risk of respiratory depression, especially when the route of administration is parenteral [13].

Ultimately, evidence from randomized controlled trials suggested a notable potential in terms of efficacy and safety (cardiovascular stability, preservation of spontaneous breathing and airway reflexes) for the use of ketamine when compared to haloperidol + midazolam/lorazepam, paving the way for further research [85, 86]. For a useful flowchart regarding the pharmacological management of PMA, the American Association of Emergency Psychiatry consensus article may be considered [13].

### 4.5 Delirium and Dementia

PMA can occur in a variety of organic conditions. In this paragraph, some main points of the current state of the art of PMA regarding delirium and dementia. These two conditions appear intertwined with delirium, leading to a greater risk of developing subsequent cognitive decline and dementia being the strongest risk factor for developing delirium [87].

Delirium is a common cause of PMA and must be promptly identified to address the specific underlying cause. A recent review shows that in delirium treatment and prevention, nonpharmacologic multicomponent approaches are the most effective strategies. Current evidence does not support the use of first- and SGAs in hospitalized older adults experiencing delirium, as they cannot alter their course [88]. However, when agitation is refractory to a nonpharmacologic approach, and symptoms expose the patients to the possibility of self-harm or harm to others, an appropriate treatment is needed. In the case of PMA associated a “start-low go-slow” approach should be followed, continuing the treatment for the minimum amount of time. Haloperidol 0.5-1mg (up to 3-5 mg per day) represents the first choice, followed by quetiapine 25-50 mg/day in patients non-suitable for the administration of haloperidol (*e.g*., Parkinson’s disease). Except for CPZ, FGAs and SGAs can be considered safe in case of liver or kidney dysfunction [36] and should be screened for pharmacokinetic interactions. Moreover, low doses of trazodone (50-300 mg/day) have also been supported in the treatment of hyperactive delirium, as long as gabapentin, pregabalin and BDZs depending on the patient’s comorbidity and general status [36].

In Alzheimer’s disease, where haloperidol is not recommended, according to available evidence, acute PMA should be treated with a low dose of risperidone as the first choice, followed by quetiapine or aripiprazole [89]. Low doses of olanzapine are supported by evidence as well [36], and recently, brexpiprazole has received FDA approval for the treatment of “Behavioural and Psychological Symptoms of Dementia,” which include agitation [90]. If a chronic treatment is needed, there’s evidence supporting the administration of citalopram [91] (up to 20 mg in over 60 patients, for possible effects on QTc and cognitive function) [92], escitalopram and sertraline [36], among others whose discussion is beyond the scope of this paragraph.

In Parkinson’s disease and Dementia with Lewy Bodies, D2-blockade should be avoided. Hence, the only antipsychotic that can be cautiously employed in the first line is quetiapine (25-75 mg/day) due to its weak D2-affinity [36]. Clozapine is also effective, but the safety profile makes this drug non-suitable for emergency settings, so the most appropriate first choice would be a short-acting BDZ (*e.g*. lorazepam) [93].

### 4.6 Pregnancy

To the best of our knowledge, no trials have compared the effectiveness and tolerability of different drugs for PMA during pregnancy. Moreover, all psychotropic medications can cross the placenta and are present in breast milk to varying degrees [94]. There is a consensus suggesting the use of haloperidol as a first-line agent [1], with no reported teratogenic effects in the second and third trimesters and limited case reports regarding the first trimester [94]. Diphenhydramine, a first-generation antihistamine, is considered safe during pregnancy according to the FDA and is a valuable choice for mild to moderate PMA. Conversely, low-potency FGAs, SGAs, BDZs, and ketamine should be avoided. As an exception among SGAs, clozapine and risperidone can be used cautiously for PMA during pregnancy, but only when necessary [94].

Therefore, it is important to prioritize DT and administer drugs at the minimum effective dosage, regardless of the drug choice. In psychiatry, agitation may occur during pregnancy as an exacerbation of a pre-existing mental disorder or as a manifestation of a new onset of illness (*e.g*., bipolar disorder, psychotic agitation), requiring careful management and treatment decisions. Untreated maternal mental illness poses significant risks to both mother and child. Hence, electroconvulsive therapy should be considered as a treatment option during pregnancy, when available [95], especially in patients with severe and uncompensated mood disorders.

### 4.7 Agitated Children and Adolescents

In 2019, a consensus from the American Association for Emergency Psychiatry was presented for the management of PMA in agitated children and adolescents. We present the indication provided by this consensus for the five most common causes of agitation in paediatric patients in Table **3** and warmly suggest the original article’s therapeutic indications for an extensive treatment of the subject tailored for clinical application [12]. It’s important to underline how children and adolescents are more vulnerable to paradoxical BDZ reactions, including BDZ-induced PMA, more frequently in autism spectrum disorders [96]. Ultimately, according to a recent systematic review, most of the available evidence supports using valproic acid, risperidone, olanzapine, ziprasidone, and aripiprazole in treating PMA in children and adolescents [96].

### 4.8 Seclusion and Restraint

Seclusion is defined as the involuntary confinement of the patient in a dedicated equipped room where he is prevented from leaving. In contrast, any form of physical immobilization of the patient can be considered as a restraint, whether it is conducted with approved mechanical holding devices (physical restraint) or through drugs administered to limit the patient's mobility (chemical restraint) [2, 97].

Forced medication, seclusion and restraint are associated with a greater risk of injuries (physical and psychological) for both patients and the staff, undermining the physician-patient relationship [97], and often representing a traumatic experience [53, 98]. Additionally, contrary to what was expected, these interventions do not lower the rate of assaults [97] and are becoming less necessary due to the therapeutic advancements in psychiatry [99]. Potential reasons for considering seclusion and restraint include confusion, lack of insight into an ongoing psychiatric condition, aggression or violence towards oneself or others, and increased risk of falling. According to the “Project BETA Seclusion and Restraint Workgroup”, these interventions can be considered only after the failure of verbal de-escalation and an attempted pharmacological intervention: restraint can be applied when the patient is a danger to the other or himself (even with hypothetic seclusion), while seclusion in a locked room can be considered if the patient would be able to regain control in an under-stimulated environment [97]. If the patient can stay quiet in an unlocked room, unlocked seclusion would be the preferred intervention [97]. During the application of restraint, a single staff member should communicate with the patients and coordinate the team. The bed should be at the lowest level possible and at a 30-degree angle [2]. The physician is required to conduct an in-person assessment within an hour [97]. Additionally, the patient's overall condition and vital signs should be monitored every 15 minutes during the first two hours, followed by hourly monitoring and repositioning of both the patient and the holding devices [2]. Both seclusion and restraint should be maintained for the shortest possible time. In all cases, the next step would be to re-engage in the physician-patient relationship with verbal de-escalation as soon as possible to reach an agreement on the next steps of the therapeutic process [97] and to discuss with the patients the reasons why seclusion or restraint was considered useful [2].

It is important to acknowledge that these interventions are not therapeutic themselves and lead, in any case, to traumatic experiences for all the parties involved. To reduce the seclusion risk, the interventions investigated were mainly preventative measures, while sensory modulation, patient and family involvement, management of PMA, and engagement in meaningful activities were applied to reduce seclusion risk [100]. Proposed restraint-reducing strategies include mechanical restraint regulations, patient-specific trigger avoidance, tailored treatment planning, and evaluation of violence and aggression to identify restraint risk factors [100, 101]. Based on the current evidence that supports the effectiveness of preventive strategies [102], there is a need for further research to identify the most important elements to target in the management of PMA. When possible, it is important to prioritize the perspective of each patient and promote the use of non-coercive therapeutic interventions to address the root causes of the emergency. Additionally, in an acute care setting, it may not be possible to follow up with patients. When coercive measures are required, this option may allow patients to elaborate on their experiences and minimize the impact on their relationship with healthcare providers [98].

## 5 CLINICAL GUIDELINES

To date, there are no comprehensive guidelines for the management of PMA in emergency and urgent care settings from a transdisciplinary perspective. However, there are various consensus documents in the literature addressing specific aspects of PMA management in several conditions. Noteworthy are the series of works conducted by the Project BETA workgroups published in 2012 [13, 15, 97, 103, 104], the more recent consensus by Marina Garriga and colleagues [1], and the protocol published by Eduard Vieta and colleagues [2]. These studies, along with numerous others concerning the management of specific conditions, primarily focus on patients with mental disorders. Regarding the management of agitation as a clinical condition, there are no shared and unified references that synthesize best clinical practices, often resulting in suboptimal treatment of patients with PMA [5]. Among the reference guidelines that come closest to meeting this need are the 2015 NICE guidelines entitled “Violence and aggression: short-term management in mental health, health, and community settings” [57]. For both pharmacological and non-pharmacological management, there are two Brazilian guidelines published by the research group led by Baldaçara *et al*. [14, 105]. Additionally, a recent clinical policy from the American College of Emergency Physicians has summarized evidence on parenteral treatments for managing adults with severe agitation [106].

**Fig. (2):**
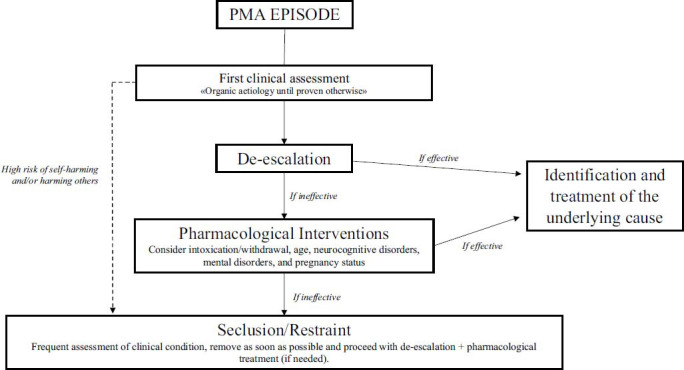
Decision-making algorithm.

PMA includes numerous conditions and clinical settings, ranging from psychiatric emergencies to intensive care units and internal medicine. Currently, PMA is referred to using interchangeable terms such as PMA and agitation. This variability reflects on one hand, the difficulty in envisioning a unified and comprehensive approach to address the needs of all involved disciplines and, on the other hand, hinders the optimal management of patients presenting with similar clinical features. Furthermore, it impedes a more in-depth and organized understanding of the phenomenon itself. Therefore, it would be desirable to focus on optimizing the assessment and treatment of patients affected by PMA in the future from a patient-centered perspective that considers both specific etiologies and a holistic epistemology (Fig. **2**).

## 6 HOSPITAL MANAGEMENT

Given that most PMA episodes are managed in emergency departments and that 46% of these cases are acquired within hospitals [107], the hospital-based management of PMA presents a significant clinical and organizational challenge due to its transnosological and nonspecific nature. Various international scientific efforts have recently identified management models and procedures to assist clinicians. The literature identifies three key elements: environmental factors, non-pharmacological measures (DT, physical restraint, or isolation), and pharmacological interventions [1]. Several controlled studies focus on pharmacological measures, while limited research on non-pharmacological interventions exists. Therefore, recommendations and expert consensus serve as the primary source of information [1, 5, 108]. These documents propose an integrated PMA management model based on risk reduction by creating a safe action environment, shared management pathways, specific healthcare staff training, and procedure monitoring and review [108]. In managing agitation, the priority should be the safety of the patient and those around them [37]. It is crucial to adopt safety protocols, provide reserved rooms to reduce external stimuli, ensure security personnel's presence, and remove dangerous objects [4]. The goal is to create a space where the patient feels physically comfortable [109].

Additionally, using appropriate verbal and non-verbal communication techniques is essential and results from the training of healthcare staff. It is essential to maintain a safe distance from the patient, avoid intense eye contact, and adopt non-threatening body positions when dealing with an agitated patient [1, 110]. Having an adequate number of staff trained in verbal DT is crucial [15]. To ensure a multidisciplinary approach, the process requires suitable clinical governance by creating hospital Diagnostic Therapeutic Assistance Pathways (DAPTs). These DAPTs should consider possible aetiologies, including risk assessment scales, and provide guidelines for pharmacological approaches and directives for using physical restraint [103, 111]. This standardised approach aims to prevent, manage, and treat agitation promptly, effectively, and collaboratively. Clinical risk management is crucial and necessitates the development of valuable indicators for monitoring. Specific staff training allows the development of skills based on clinical data and evidence, improving coordination among staff and thereby increasing intervention effectiveness [1, 112]. From a clinical perspective, experts recommend intervening, preferably when symptoms are low-moderate or moderate severity, to reduce the risk of worsening, enhance patient collaboration, and achieve better clinical outcomes [5]. Interventions should begin with non-pharmacological measures [14], such as environmental modifications and verbal de-escalation. Professional attitudes, such as a calm, empathetic, and respectful approach, are fundamental in managing agitation [113-115]. Non-pharmacological interventions are considered essential at all levels of PMA, whether used alone in mild cases or conjunction with pharmacological interventions [108]. However, a thorough assessment of agitation causes is necessary before pharmacological interventions.

Verbal techniques have been shown to reduce agitation and the risk of associated violence. It is advisable to intervene verbally or administer medication with the patient's consent before resorting to more invasive strategies [116]. Physical restraint should be the last resort and used temporarily to prevent negative consequences [117]. The Joint Commission on Accreditation of Healthcare Organizations (JCAHO) recommends its use only in emergencies where other management attempts have failed. Moreover, it should not be used as a punishment or for staff convenience [109, 110]. In any case, verbal de-escalation attempts should continue, and medication should be administered to calm a patient in restraint, monitoring the response and preventing complications. In a multidisciplinary approach, expert consensus also emphasizes the inclusion of other healthcare professionals in the integrated model: the nurse as a case manager (CM) and the hospital pharmacist. The nurse plays a central role in personalising care and providing support to the patient, which can be particularly effective in the emergency department with adequate training in psychiatric conditions [108, 118]. The hospital pharmacist, conversely, can be included in the context of “pharmaceutical care” [108, 119]. They can be responsible for selecting drugs for the formulary, evaluating the clinical profile and cost-effectiveness, and contributing to safe and effective drug use. In order to improve and standardise the approach to patients with PMA, monitoring is crucial. Clinical data from hospital registries help update procedures and guidelines and assess their effectiveness.

Additionally, PMA episodes lead to increased healthcare costs and resource utilisation, putting patients and healthcare personnel at risk [10, 120, 121]. However, there is a lack of pharmacoeconomic data on the consequences of violent behaviour in patients with PMA. Therefore, to rationalise available economic resources, it is desirable to collect data on direct costs, such as hospital length of stay and pharmaceutical expenses [10], and indirect non-healthcare costs associated with the consequences of aggressive behaviours. In conclusion, early identification, appropriate management, and pharmacological and non-pharmacological treatment of agitation are necessary, and their improvement can positively impact the experience and safety of patients and medical personnel while optimising healthcare resource utilisation. In order to achieve that end, the use of protocols, a multidisciplinary approach, and continuous training and monitoring activities are highly recommended [6, 122].

## CONCLUSION

PMA represents a syndrome that can stem from a wide number of medical conditions and can be present in many different disciplines. These features account for the fact that available evidence is somehow scattered and limited to specific populations. It is frequently treated in hospital settings, often under emergency conditions, and the clinical management requires clear and accessible guidelines applicable easily across all areas.

The foundational approach for the management of an agitated patient must be based on DT provided in a secure environment. In all cases, the phenomenon must be considered organic until proven otherwise, especially in over 45 years old patients. An initial clinical evaluation should be conducted swiftly, based on the available medical history and a physical examination, aiming at identifying the key elements that can differentiate the aetiology of the current episode. A routine laboratory evaluation and the assessment of vital parameters should always be performed.

If verbal de-escalation fails, pharmacological strategies with a calming rather than sedative action are indicated, with the ultimate purpose of achieving the best therapeutic alliance possible. The choice of medication should consider the patient's overall condition, any ongoing therapies, age, signs or symptoms of alcohol/substance intoxication, and pregnancy status. The route of administration should preferably be non-invasive and chosen in agreement with the patient whenever possible. When such an approach fails or is impossible to apply due to a state of necessity, seclusion and restraint can be used for the shortest time, following available protocols and observing strict clinical supervision.

The management of PMA encompasses a wide range of areas and different clinical conditions, which hinders the development of unified approaches. However, due to its complexity and often high risk, it is essential to unify research in this field from a transdisciplinary perspective. Moreover, further study should clarify the possible role of specific assessment tools in PMA decisional protocols. Such efforts would support the clinical decisions of healthcare professionals, enabling optimized management to benefit both patients and healthcare workers.

## Figures and Tables

**Table 1:** Overview of the key clinical elements to consider in the patient with PMA in three steps.

**1** **Initial Approach in Case of a PMA Episode** • Adopt a de-escalation-based approach to the patient. • Establish a safe clinical environment. • Provide a quick general medical status assessment (clinical history/current therapy).		
Signs/Symptoms requiring further assessment		Neurologic signs/symptoms
		Headache
		Cardiopulmonary signs/symptoms
		Abdominal symptoms (*e.g*. nausea, vomiting)
		Evidence of trauma/recent surgery
		Evidence of toxidrome
		Metabolic/Endocrine alterations (*e.g*., glucose levels, cortisol levels, electrolytes, malnutrition)
		Evidence of substance withdrawal
		Involuntary weight loss
		Heat intolerance, Hyperthermia
		Infection (*e.g*. fever, cutaneous rash)
		New-onset psychosis
**2** **Differential Diagnosis**		
**Possible Organic Causes**		**Possible Correlated Mental Disorders**
Trauma • Burns • Traumatic brain injury Infection • Meningitidis/Encephalitis • Sepsis • Syphilis • HIV Toxicologic cause • Serotonin syndrome • Neuroleptic Malignant Syndrome • Withdrawal/Intoxication • Steroid-induced psychosis • Antipsychotics - (*e.g*. akathisia) Respiratory • Respiratory failure • Hypoxia • Hypercarbia Cardiovascular • Shock • Hypertensive encephalopathy Metabolic/Endocrine • Thyroid disorders (*e.g*., Graves’ Disease) • Nutritional Deficiency (*e.g*. Wernicke-Korsakoff syndrome) Neurologic • Stroke • Vasculitis • Haemorrhage • Hydrocephalus • Dementia • Seizure - postictal agitation • Huntington’s Disease • Multiple Sclerosis Oncologic • Paraneoplastic syndromes		Psychotic disorders • Schizophrenia spectrum disorders Mood disorders • Manic episodes, depressive episodes with mixed features, and mixed states (bipolar disorder) • Agitated depression (“unipolar” depression) Anxiety disorders Personality disorders • Borderline personality disorder Reactive or situational agitation Neurodevelopmental disorders • Autism spectrum disorder • Intellectual disability
**3** **Assessment/Treatment (see dedicated section 3/ Table ** **3** **)**		

**Table 2:** Summary of available scales to evaluate agitation.

**Tool**	**Principal Setting**	**Further Application Settings**
ABS	Long-term care facilities	Acute care
CABS	Acquired brain injury acute phase	Emergency departments
BARS	Nursing homes	Psychiatric services
BVC^§^	Psychiatric services	-
CGI-A	Psychiatric services	-
CMAI	Elderly patients in long-term care facilities	Psychiatric services
HCR-20* ^§^	Wide range of clinical, forensic, correctional and research settings	-
VSC^§^	Psychiatric services	-
NRS-R	Patients with neurobehavioural disturbances	Emergency departments
OAS	Adult and paediatric patients, clinical and research settings	-
OASS	Psychiatric inpatients	-
PANSS-EC*	Assessment of mental disorders	Clinical trials of pharmacotherapy for agitation
RAS	Nursing homes	-
ACES	Psychiatric clinical and research settings	-

**Abbreviations**: ABS: Aggressive Behavior Scale; ACES: Agitation-Calmness Evaluation Scale; BARS: Brief Agitation Rating Scale; BVC: Brøset Violence Checklist; CABS: Corrigan Agitated Behavior Scale; CGI-A: Clinical Global Impression Scale for Aggression; CMAI: Cohen-Mansfield Agitation Inventory; HCR-20: Historical, Clinical, and Risk Management-20 Violence Risk Assessment Scheme; NRS-R: Neurobehavioral Rating Scale-Revised; OAS: Overt Aggression Scale; OASS: Overt Agitation Severity Scale; PANSS-EC: Positive and Negative Syndrome Scale-Excited Com-ponent; RAS: Ryden Aggression Scale; SAS: Sedation-Agitation Scale; VSC: McNiel-Binder Violence Screening Checklist.

*correlated with the need for medication, ^§^prediction of aggression/violence.

**Table 3:** Therapeutic options recommended for the treatment of PMA in children and adolescents [12].

**Condition Underlying PMA**	**Recommendation**
Delirium	*SGAs - first-line agents* -Risperidone -Olanzapine -Quetiapine *FGAs* -CPZ -Haloperidol *Other* -Clonidine (if administration of neuroleptic is impossible) -Melatonin (if circadian rhythm disruption)
Substance intoxication/withdrawal (if unknown substance)	-Lorazepam -Lorazepam + haloperidol (if psychotic symptoms)
Autism spectrum disorder	(1) Extra dose of the regular standing medication* (2) avoid BDZs due to the risk of disinhibition. -Clonidine -Diphenhydramine -Risperidone/ CPZ /olanzapine
Unknown aetiology	*Mild PMA* DT *Moderate PMA* -Lorazepam -Diphenhydramine -Olanzapine *Severe PMA* -Lorazepam + haloperidol - CPZ -Olanzapine
Mental Disorder	ADHD -Clonidine -Diphenhydramine -Risperidone Agitated catatonia -Lorazepam Anxiety disorders or Trauma-related/Stress-related disorders -Lorazepam -Clonidine Oppositional defiant disorder/Conduct disorder -CPZ -Lorazepam -Olanzapine -Risperidone *Psychosis/Bipolar Disorder* -Extra dose of standing medication (first line) -Risperidone -Quetiapine -CPZ -Lorazepam -Lorazepam + haloperidol

**Note**: *Patients affected by autism spectrum disorders often are vulnerable to adverse side effects from medications commonly used to treat agitation, new treatments should be introduced with caution. **Abbreviations**: ADHD: Attention-Deficit/Hyperactivity Disorder; BDZs: Benzodiazepines; CPZ: Chlorpromazine; PMA: Psychomotor agitation; SGAs: Second-generation Antipsychotics.

## LIST OF ABBREVIATIONS

ABS: Aggressive Behavior Scale
ACES: Agitation-calmness Evaluation Scale
BARS: Brief Agitation Rating Scale
BDZ[s]: Benzodiazepine[s]
BVC: Brøset Violence Checklist
CABS: Corrigan Agitated Behavior Scale
CGI-A: Clinical Global Impression Scale for Aggression
CMAI: Cohen-mansfield Agitation Inventory
CPZ: Chlorpromazine
DAPTs: Diagnostic Therapeutic Assistance Pathways
DT: De-escalation Techniques
FGA[s]: First-generation Antipsychotic[s]
FT3: Triiodothyronine
HCR-20: Historical, Clinical, and Risk Management-20 Violence Risk Assessment Scheme
IL-1b: Interleukin-1 beta
NRS-R: Neurobehavioral Rating Scale-revised
OAS: Overt Aggression Scale
OASS: Overt Agitation Severity Scale
PANSS-EC: Positive and Negative Syndrome Scale-excited Component
PMA: Psychomotor Agitation
RAS: Ryden Aggression Scale
SAS: Sedation-agitation Scale
SGA[s]: Second-generation Antipsychotic[s]
TSH: Thyroid-stimulating Hormone
VSC: McNiel-Binder Violence Screening Checklist

## References

[1] Garriga M., Pacchiarotti I., Kasper S., Zeller S.L., Allen M.H., Vázquez G., Baldaçara L., San L., McAllister-Williams R.H., Fountoulakis K.N., Courtet P., Naber D., Chan E.W., Fagiolini A., Möller H.J., Grunze H., Llorca P.M., Jaffe R.L., Yatham L.N., Hidalgo-Mazzei D., Passamar M., Messer T., Bernardo M., Vieta E. (2016). Assessment and management of agitation in psychiatry: Expert consensus.. World J. Biol. Psychiatry.

[2] Vieta E., Garriga M., Cardete L., Bernardo M., Lombraña M., Blanch J., Catalán R., Vázquez M., Soler V., Ortuño N., Martínez-Arán A. (2017). Protocol for the management of psychiatric patients with psychomotor agitation.. BMC Psychiatry.

[3] Luchowski P., Sojka M., Oleksak I., Jartych A., Piwoński M., Rejdak K. (2022). Acute psychomotor agitation – Challenges for psychiatrists and neurologists: A case study.. Postepy Psychiatr. Neurol..

[4] Pompili M., Ducci G., Galluzzo A., Rosso G., Palumbo C., De Berardis D. (2021). The management of psychomotor agitation associated with schizophrenia or bipolar disorder: A brief review.. Int. J. Environ. Res. Public Health.

[5] Martínez-Raga J., Amore M., Di Sciascio G., Florea R.I., Garriga M., Gonzalez G., Kahl K.G., Karlsson P.A., Kuhn J., Margariti M., Pacciardi B., Papageorgiou K., Pompili M., Rivollier F., Royuela Á., Safont G., Scharfetter J., Skagen B., Tajima-Pozo K., Vidailhet P. (2018). 1st International experts’ meeting on agitation: Conclusions regarding the current and ideal management paradigm of agitation.. Front. Psychiatry.

[6] San L., Marksteiner J., Zwanzger P., Figuero M.A., Romero F.T., Kyropoulos G., Peixoto A.B., Chirita R., Boldeanu A. (2016). State of acute agitation at psychiatric emergencies in Europe: The STAGE study.. Clin. Pract. Epidemiol. Ment. Health.

[7] Gottlieb M., Long B., Koyfman A. (2018). Approach to the agitated emergency department patient.. J. Emerg. Med..

[8] Accinni T., Frascarelli M., Vecchioni S., Tarsitani L., Biondi M., Pasquini M. (2022). Gender modulation of psychopathological dimensions associated to suicidality.. Riv. Psichiatr..

[9] da Silva A.G., Baldaçara L., Cavalcante D.A., Fasanella N.A., Palha A.P. (2020). The impact of mental illness stigma on psychiatric emergencies.. Front. Psychiatry.

[10] Rubio-Valera M., Luciano J.V., Ortiz J.M., Salvador-Carulla L., Gracia A., Serrano-Blanco A. (2015). Health service use and costs associated with aggressiveness or agitation and containment in adult psychiatric care: A systematic review of the evidence.. BMC Psychiatry.

[11] Day R.K. (1999). Psychomotor agitation: Poorly defined and badly measured.. J. Affect. Disord..

[12] Gerson R., Malas N., Feuer V., Silver G., Prasad R., Mroczkowski M., Pena-Nowak M., Gaveras G., Goepfert E., Hartselle S., Henderson S., Jhonsa A., Kelly P., Mangini L., Maxwell B., Prager L., Prasad R., DePena-Nowak M. (2019). Best practices for evaluation and treatment of agitated children and adolescents (BETA) in the emergency department: Consensus statement of the american association for emergency psychiatry.. West. J. Emerg. Med..

[13] Wilson M., Pepper D., Currier G., Holloman G., Feifel D. (2012). The psychopharmacology of agitation: Consensus statement of the american association for emergency psychiatry project Beta psychopharmacology workgroup.. West. J. Emerg. Med..

[14] Baldaçara L., Ismael F., Leite V., Pereira L.A., dos Santos R.M., Gomes Júnior V.P., Calfat E.L.B., Diaz A.P., Périco C.A.M., Porto D.M., Zacharias C.E., Cordeiro Q., da Silva A.G., Tung T.C. (2019). Brazilian guidelines for the management of psychomotor agitation. Part 1. Non-pharmacological approach.. Br. J. Psychiatry.

[15] Richmond J., Berlin J., Fishkind A., Holloman G., Zeller S., Wilson M., Rifai M.A., Ng A. (2012). Verbal de-escalation of the agitated patient: Consensus statement of the American association for emergency psychiatry project BETA De-escalation workgroup.. West. J. Emerg. Med..

[16] Downing L.J., Caprio T.V., Lyness J.M. (2013). Geriatric psychiatry review: Differential diagnosis and treatment of the 3 D’s - delirium, dementia, and depression.. Curr. Psychiatry Rep..

[17] Oh S.T., Park J.Y. (2019). Postoperative delirium.. Korean J. Anesthesiol..

[18] Fong T.G., Inouye S.K. (2022). The inter-relationship between delirium and dementia: The importance of delirium prevention.. Nat. Rev. Neurol..

[19] Woodford H.J., George J., Jackson M. (2015). Non-convulsive status epilepticus: A practical approach to diagnosis in confused older people.. Postgrad. Med. J..

[20] Martinotti G., Negri A., Schiavone S., Montemitro C., Vannini C., Baroni G., Pettorruso M., De Giorgio F., Giorgetti R., Verrastro V., Trabace L., Garcia A., Castro I., Iglesias Lopez J., Merino Del Villar C., Schifano F., di Giannantonio M. (2020). Club drugs: Psychotropic effects and psychopathological characteristics of a sample of inpatients.. Front. Psychiatry.

[21] Mirijello A., Sestito L., Antonelli M., Gasbarrini A., Addolorato G. (2023). Identification and management of acute alcohol intoxication.. Eur. J. Intern. Med..

[22] George C., Jacob T.R., Kumar A.V. (2016). Pattern and correlates of agitation in an acute psychiatry in-patient setting in a teaching hospital.. Asian J. Psychiatr..

[23] Hankin C.S., Bronstone A., Koran L.M. (2011). Agitation in the inpatient psychiatric setting: A review of clinical presentation, burden, and treatment.. J. Psychiatr. Pract..

[24] Roppolo L.P., Morris D.W., Khan F., Downs R., Metzger J., Carder T., Wong A.H., Wilson M.P. (2020). Improving the management of acutely agitated patients in the emergency department through implementation of Project BETA (Best Practices in the Evaluation and Treatment of Agitation).. J. Am. Coll. Emerg. Physicians Open.

[25] Katus L.E., Frucht S.J. (2016). Management of serotonin syndrome and neuroleptic malignant syndrome.. Curr. Treat. Options Neurol..

[26] Melis G., Pia G., Piras I., Tusconi M. (2015). Mental disorders and HIV infection in the emergency department: Epidemiology and gender differences.. Intern. Emerg. Med..

[27] Sampogna G., Del Vecchio V., Giallonardo V., Luciano M., Fiorillo A. (2020). Diagnosis, clinical features, and therapeutic implications of agitated depression.. Psychiatr. Clin. North Am..

[28] Salem H., Nagpal C., Pigott T., Teixeira A.L. (2017). Revisiting antipsychotic-induced akathisia: Current issues and prospective challenges.. Curr. Neuropharmacol..

[29] Shaikh U., Qamar I., Jafry F., Hassan M., Shagufta S., Odhejo Y.I., Ahmed S. (2017). Patients with borderline personality disorder in emergency departments.. Front. Psychiatry.

[30] Hamasaki H., Yoshimi T., Yanai H. (2012). A patient with Graves’ disease showing only psychiatric symptoms and negativity for both TSH receptor autoantibody and thyroid stimulating antibody.. Thyroid Res..

[31] Lawlor B., Ni Bhriain S. (2001). Psychosis and behavioural symptoms of dementia: Defining the role of neuroleptic interventions.. Int. J. Geriatr. Psychiatry.

[32] Dabir A., Zahoor S., Abou-Khalil B.W. (2022). Determinants of postictal agitation and recovery after tonic‐clonic seizures in generalized and focal epilepsy.. Epileptic Disord..

[33] Diaz-Olavarrieta C., Cummings J.L., Velazquez J., Garcia de al Cadena C. (1999). Neuropsychiatric manifestations of multiple sclerosis.. J. Neuropsychiatry Clin. Neurosci..

[34] Yoshino T., Yamamoto R., Hoshina Y., Ishimine T. (2023). Anti-SOX-1 antibody-positive paraneoplastic limbic encephalitis diagnosed during small cell lung cancer treatment.. Cureus.

[35] Bertsch K., Florange J., Herpertz S.C. (2020). Understanding brain mechanisms of reactive aggression.. Curr. Psychiatry Rep..

[36] Carrarini C., Russo M., Dono F., Barbone F., Rispoli M.G., Ferri L., Di Pietro M., Digiovanni A., Ajdinaj P., Speranza R., Granzotto A., Frazzini V., Thomas A., Pilotto A., Padovani A., Onofrj M., Sensi S.L., Bonanni L. (2021). Agitation and dementia: Prevention and treatment strategies in acute and chronic conditions.. Front. Neurol..

[37] Miller J. (2021). Managing acute agitation and aggression in the world of drug shortages.. Ment. Health Clin..

[38] Li C., Shi Z., Ji J., Niu G., Liu Z. (2021). Associations of C-reactive protein, free triiodothyronine, thyroid stimulating hormone and creatinine levels with agitation in patients with schizophrenia: A comparative cross-sectional study.. Neuropsychiatr. Dis. Treat..

[39] Higuchi M., Hatta K., Honma T., Hitomi Y.H., Kambayashi Y., Hibino Y., Matsuzaki I., Sasahara S., Nakamura H. (2010). Association between altered systemic inflammatory interleukin‐1β and natural killer cell activity and subsequently agitation in patients with alzheimer disease.. Int. J. Geriatr. Psychiatry.

[40] Zeller S.L., Rhoades R.W. (2010). Systematic reviews of assessment measures and pharmacologic treatments for agitation.. Clin. Ther..

[41] Pereira L.A., da Silva A.G., Hemanny C., de Jesus R., Moromizato M., Vieira T., Souza M., Lima M.G., Baldaçara L. (2023). Translation, cross-cultural adaptation, and validation of the behavioral activity rating scale (BARS) for the Brazilian population.. Trends Psychiatry Psychother..

[42] Garrote-Cámara M.E., Santolalla-Arnedo I., Ruiz de Viñaspre-Hernández R., Gea-Caballero V., Sufrate-Sorzano T., del Pozo-Herce P., Garrido-García R., Rubinat-Arnaldo E., Juárez Vela R. (2021). Psychometric characteristics and sociodemographic adaptation of the corrigan agitated behavior scale in patients with severe mental disorders.. Front. Psychol..

[43] Garrote-Cámara M.E., Gea-Caballero V., Sufrate-Sorzano T., Rubinat-Arnaldo E., Santos-Sánchez J.Á., Cobos-Rincón A., Santolalla-Arnedo I., Juárez-Vela R. (2022). Clinical and sociodemographic profile of psychomotor agitation in mental health hospitalisation: A multicentre study.. Int. J. Environ. Res. Public Health.

[44] De Filippis S., Cuomo I., Lionetto L., Janiri D., Simmaco M., Caloro M., De Persis S., Piazzi G., Simonetti A., Telesforo C.L., Sciarretta A., Caccia F., Gentile G., Kotzalidis G.D., Girardi P. (2013). Intramuscular aripiprazole in the acute management of psychomotor agitation.. Pharmacotherapy.

[45] Suzuki H., Gen K., Takahashi Y. (2014). A naturalistic comparison study of the efficacy and safety of intramuscular olanzapine, intramuscular haloperidol, and intramuscular levomepromazine in acute agitated patients with schizophrenia.. Hum. Psychopharmacol..

[46] Zhang S., Cui W., Ding S., Li X., Zhang X.W., Wu Y. (2024). A cluster-randomized controlled trial of a nurse-led artificial intelligence assisted prevention and management for delirium (AI-AntiDelirium) on delirium in intensive care unit: Study protocol.. PLoS One.

[47] Pacciardi B., Mauri M., Cargioli C., Belli S., Cotugno B., Di Paolo L., Pini S. (2013). Issues in the management of acute agitation: How much current guidelines consider safety?. Front. Psychiatry.

[48] Weltens I., Bak M., Verhagen S., Vandenberk E., Domen P., van Amelsvoort T., Drukker M. (2021). Aggression on the psychiatric ward: Prevalence and risk factors. A systematic review of the literature.. PLoS One.

[49] Hallett N., Dickens G.L. (2017). De-escalation of aggressive behaviour in healthcare settings: Concept analysis.. Int. J. Nurs. Stud..

[50] Kaplan S.G., Wheeler E.G. (1983). Survival skills for working with potentially violent clients.. Soc. Casework.

[51] Maier G.J. (1996). Managing threatening behavior. The role of talk down and talk up.. J. Psychosoc. Nurs. Ment. Health Serv..

[52] Butterworth H., Wood L., Rowe S. (2022). Patients’ and staff members’ experiences of restrictive practices in acute mental health in-patient settings: Systematic review and thematic synthesis.. BJPsych Open.

[53] Chieze M., Hurst S., Kaiser S., Sentissi O. (2019). Effects of seclusion and restraint in adult psychiatry: A systematic review.. Front. Psychiatry.

[54] Muralidharan S., Fenton M. (2006). Containment strategies for people with serious mental illness.. Cochrane Libr..

[55] Du M., Wang X., Yin S., Shu W., Hao R., Zhao S., Rao H., Yeung W.L., Jayaram M.B., Xia J. (2017). De-escalation techniques for psychosis-induced aggression or agitation.. Cochrane Libr..

[56] Kuivalainen S., Vehviläinen-Julkunen K., Louheranta O., Putkonen A., Repo-Tiihonen E., Tiihonen J. (2017). De‐escalation techniques used, and reasons for seclusion and restraint, in a forensic psychiatric hospital.. Int. J. Ment. Health Nurs..

[57] (2015). Violence and aggression: short-term management in mental health, health and community settings.

[58] Kleespies P.M., Richmond J.S., Kleespies P.M. (2009). Evaluating behavioral emergencies: The clinical interview.. Behavioral emergencies: An evidence-based resource for evaluating and managing risk of suicide, violence, and victimization..

[59] Bowers L. (2014). A model of de-escalation.. Ment. Health Pract..

[60] Berring L.L., Pedersen L., Buus N. (2016). Coping with violence in mental health care settings: Patient and staff member perspectives on de-escalation practices.. Arch. Psychiatr. Nurs..

[61] Völlm B.A., Taylor A.N.W., Richardson P., Corcoran R., Stirling J., McKie S., Deakin J.F.W., Elliott R. (2006). Neuronal correlates of theory of mind and empathy: A functional magnetic resonance imaging study in a nonverbal task.. Neuroimage.

[62] Valentini M., Pinucci I., Pasquini M., Biondi M., Pasquini M., Tarsitani L. (2021). Empathy Regulation in Crisis Scenario.. Empathy, Normalization and De-escalation..

[63] Holloman G., Zeller S. (2012). Overview of project BETA: Best practices in evaluation and treatment of agitation.. West. J. Emerg. Med..

[64] Accinni T., Papadogiannis G., Orso L., Biondi M., Pasquini M., Tarsitani L. (2021). De-escalation Techniques in Various Settings.. Empathy, Normalization and De-escalation..

[65] Egan G. (1975). The Skilled Helper: A Systematic Approach to Effective Helping..

[66] Stickley T. (2011). From soler to surety for effective non-verbal communication.. Nurse Educ. Pract..

[67] Spencer S., Johnson P., Smith I.C. (2018). De-escalation techniques for managing non-psychosis induced aggression in adults.. Cochrane Libr..

[68] Brenig D., Gade P., Voellm B. (2023). Is mental health staff training in de-escalation techniques effective in reducing violent incidents in forensic psychiatric settings? – A systematic review of the literature.. BMC Psychiatry.

[69] Stubbe D.E. (2023). Psychiatric emergencies: Empowering connections to de-escalate aggression.. Focus Am. Psychiatr. Publ..

[70] Cole J.B., Klein L.R., Martel M.L. (2019). Parenteral antipsychotic choice and its association with emergency department length of stay for acute agitation secondary to alcohol intoxication.. Acad. Emerg. Med..

[71] Muncie H.L., Yasinian Y., Oge’ L. (2013). Outpatient management of alcohol withdrawal syndrome.. Am. Fam. Physician.

[72] Tidwell W.P., Thomas T.L., Pouliot J.D., Canonico A.E., Webber A.J. (2018). Treatment of alcohol withdrawal syndrome: Phenobarbital vs. CIWA-Ar protocol.. Am. J. Crit. Care.

[73] Malone D., Costin B.N., MacElroy D., Al-Hegelan M., Thompson J., Bronshteyn Y. (2023). Phenobarbital versus benzodiazepines in alcohol withdrawal syndrome.. Neuropsychopharmacol. Rep..

[74] Day E., Daly C. (2022). Clinical management of the alcohol withdrawal syndrome.. Addiction.

[75] Isbister G.K., Calver L.A., Page C.B., Stokes B., Bryant J.L., Downes M.A. (2010). Randomized controlled trial of intramuscular droperidol versus midazolam for violence and acute behavioral disturbance: the DORM study.. Ann. Emerg. Med..

[76] Pepa P.A., Lee K.C., Huynh H.E., Wilson M.P. (2017). Safety of risperidone for acute agitation and alcohol intoxication in emergency department patients.. J. Emerg. Med..

[77] Mullinax S., Shokraneh F., Wilson M.P., Adams C.E. (2017). Oral medication for agitation of psychiatric origin: A scoping review of randomized controlled trials.. J. Emerg. Med..

[78] Hirsch S., Steinert T. (2019). The use of rapid tranquilization in aggressive behavior.. Dtsch. Arztebl. Int..

[79] Ahmed U., Jones H., Adams C.E. (2010). Chlorpromazine for psychosis induced aggression or agitation.. Cochrane Libr..

[80] Citrome L., Volavka J. (1999). Violent patients in the emergency setting.. Psychiatr. Clin. North Am..

[81] McDowell M., Nitti K., Kulstad E., Cirone M., Shah R., Rochford D., Walsh R., Hesse K. (2019). Clinical outcomes in patients taking inhaled loxapine, haloperidol, or ziprasidone in the emergency department.. Clin. Neuropharmacol..

[82] Battaglia J. (2005). Pharmacological management of acute agitation.. Drugs.

[83] Jeffers T., Darling B., Edwards C., Vadiei N. (2022). Efficacy of combination haloperidol, lorazepam, and diphenhydramine vs. combination haloperidol and lorazepam in the treatment of acute agitation: A multicenter retrospective cohort study.. J. Emerg. Med..

[84] Williams A.M. (2018). Coadministration of intramuscular olanzapine and benzodiazepines in agitated patients with mental illness.. Ment. Health Clin..

[85] Barbic D., Andolfatto G., Grunau B., Scheuermeyer F.X., Macewan B., Qian H., Wong H., Barbic S.P., Honer W.G. (2021). Rapid agitation control with ketamine in the emergency department: A blinded, randomized controlled trial.. Ann. Emerg. Med..

[86] Lin J., Figuerado Y., Montgomery A., Lee J., Cannis M., Norton V.C., Calvo R., Sikand H. (2021). Efficacy of ketamine for initial control of acute agitation in the emergency department: A randomized study.. Am. J. Emerg. Med..

[87] Maldonado J.R. (2018). Delirium pathophysiology: An updated hypothesis of the etiology of acute brain failure.. Int. J. Geriatr. Psychiatry.

[88] Oh E.S., Fong T.G., Hshieh T.T., Inouye S.K. (2017). Delirium in older persons.. JAMA.

[89] Davies S.J.C., Burhan A.M., Kim D., Gerretsen P., Graff-Guerrero A., Woo V.L., Kumar S., Colman S., Pollock B.G., Mulsant B.H., Rajji T.K. (2018). Sequential drug treatment algorithm for agitation and aggression in Alzheimer’s and mixed dementia.. J. Psychopharmacol..

[90] Varadharajan A., Davis A.D., Ghosh A., Jagtap T., Xavier A., Menon A.J., Roy D., Gandhi S., Gregor T. (2023). Guidelines for pharmacotherapy in Alzheimer’s disease - A primer on FDA-approved drugs.. J. Neurosci. Rural Pract..

[91] Porsteinsson A.P., Drye L.T., Pollock B.G., Devanand D.P., Frangakis C., Ismail Z., Marano C., Meinert C.L., Mintzer J.E., Munro C.A., Pelton G., Rabins P.V., Rosenberg P.B., Schneider L.S., Shade D.M., Weintraub D., Yesavage J., Lyketsos C.G. (2014). Effect of citalopram on agitation in Alzheimer disease: The CitAD randomized clinical trial.. JAMA.

[92] Porsteinsson A.P., Smith J.S., Keltz M.A., Antonsdottir I.M. (2014). Can antidepressant medication relieve agitation in Alzheimer’s disease?. Expert Rev. Neurother..

[93] Jones S.L., Hindle J.V. (2011). Parkinson’s disease in the acute hospital.. Clin. Med..

[94] Gartenberg A., Levine K., Petrie A. (2024). Emergency department management of acute agitation in the reproductive age female and pregnancy.. World J. Emerg. Med..

[95] Ward H.B., Fromson J.A., Cooper J.J., De Oliveira G., Almeida M. (2018). Recommendations for the use of ECT in pregnancy: Literature review and proposed clinical protocol.. Arch. Women Ment. Health.

[96] Tripodi B., Matarese I., Carbone M.G. (2023). A critical review of the psychomotor agitation treatment in youth.. Life.

[97] Knox D., Holloman G. (2012). Use and avoidance of seclusion and restraint: Consensus statement of the american association for emergency psychiatry project Beta seclusion and restraint workgroup.. West. J. Emerg. Med..

[98] Larue C., Dumais A., Boyer R., Goulet M.H., Bonin J.P., Baba N. (2013). The experience of seclusion and restraint in psychiatric settings: Perspectives of patients.. Issues Ment. Health Nurs..

[99] Masters K.J., Huckshorn K.A. (2020). The role of the psychiatrist in seclusion and restraint.. Psychiatr. Serv..

[100] Väkiparta L., Suominen T., Paavilainen E., Kylmä J. (2019). Using interventions to reduce seclusion and mechanical restraint use in adult psychiatric units: An integrative review.. Scand. J. Caring Sci..

[101] Simpson S.A., Joesch J.M., West I.I., Pasic J. (2014). Risk for physical restraint or seclusion in the psychiatric emergency service (PES).. Gen. Hosp. Psychiatry.

[102] Blair E.W., Woolley S., Szarek B.L., Mucha T.F., Dutka O., Schwartz H.I., Wisniowski J., Goethe J.W. (2017). Reduction of seclusion and restraint in an inpatient psychiatric setting: A pilot study.. Psychiatr. Q..

[103] Nordstrom K., Zun L., Wilson M., Stiebel V., Ng A., Bregman B., Anderson E. (2012). Medical evaluation and triage of the agitated patient: Consensus statement of the american association for emergency psychiatry project Beta medical evaluation workgroup.. West. J. Emerg. Med..

[104] Stowell K., Florence P., Harman H., Glick R. (2012). Psychiatric evaluation of the agitated patient: Consensus statement of the american association for emergency psychiatry project Beta psychiatric evaluation workgroup.. West. J. Emerg. Med..

[105] Baldaçara L., Diaz A.P., Leite V., Pereira L.A., dos Santos R.M., Gomes Júnior V.P., Calfat E.L.B., Ismael F., Périco C.A.M., Porto D.M., Zacharias C.E.K., Cordeiro Q., da Silva A.G., Tung T.C. (2019). Brazilian guidelines for the management of psychomotor agitation. Part 2. Pharmacological approach.. Br. J. Psychiatry.

[106] Thiessen M.E.W., Godwin S.A., Hatten B.W., Whittle J.A., Haukoos J.S., Diercks D.B., Diercks D.B., Wolf S.J., Anderson J.D., Byyny R., Carpenter C.R., Friedman B., Gemme S.R., Gerardo C.J., Godwin S.A., Hahn S.A., Hatten B.W., Haukoos J.S., Kaji A., Kwok H., Lo B.M., Mace S.E., Moran M., Promes S.B., Shah K.H., Shih R.D., Silvers S.M., Slivinski A., Smith M.D., Thiessen M.E.W., Tomaszewski C.A., Valente J.H., Wall S.P., Westafer L.M., Yu Y., Cantrill S.V., Finnell J.T., Schulz T., Vandertulip K. (2024). Clinical policy: Critical issues in the evaluation and management of adult out-of-hospital or emergency department patients presenting with severe agitation: Approved by the ACEP board of directors, October 6, 2023.. Ann. Emerg. Med..

[107] Mansutti I., Venturini M., Palese A. (2020). Episodes of psychomotor agitation among medical patients: Findings from a longitudinal multicentre study.. Aging Clin. Exp. Res..

[108] Gruppo A.I.R. (2016). Consensus document: a model of integrated management of patients with psycomotor agitation.. Riv. Psichiatr..

[109] Marder S.R. (2006). A review of agitation in mental illness: Treatment guidelines and current therapies.. J. Clin. Psychiatry.

[110] Petit J.R. (2005). Management of the acutely violent patient.. Psychiatr. Clin. North Am..

[111] De Fruyt J., Demyttenaere K. (2004). Rapid tranquilization: New approaches in the emergency treatment of behavioral disturbances.. Eur. Psychiatry.

[112] Wong A.H.W., Combellick J., Wispelwey B.A., Squires A., Gang M. (2017). The patient care paradox: An interprofessional qualitative study of agitated patient care in the emergency department.. Acad. Emerg. Med..

[113] Mantovani C., Migon M.N., Alheira F.V., Del-Ben C.M. (2010). Management of the violent or agitated patient.. Braz. J. Psychiatry.

[114] Jensen L., Clough R. (2016). Assessing and treating the patient with acute psychotic disorders.. Nurs. Clin. North Am..

[115] McCann T.V., Baird J., Muir-Cochrane E. (2014). Attitudes of clinical staff toward the causes and management of aggression in acute old age psychiatry inpatient units.. BMC Psychiatry.

[116] Allen M.H., Currier G.W., Hughes D.H., Reyes-Harde M., Docherty J.P. (2001). The expert consensus guideline series. Treatment of behavioral emergencies.. Postgrad. Med..

[117] Reitan S.K., Helvik A.S., Iversen V. (2018). Use of mechanical and pharmacological restraint over an eight-year period and its relation to clinical factors.. Nord. J. Psychiatry.

[118] Turner S.B., Stanton M.P. (2015). Psychiatric case management in the emergency department.. Prof. Case Manag..

[119] Allemann S.S., van Mil J.W.F., Botermann L., Berger K., Griese N., Hersberger K.E. (2014). Pharmaceutical care: The PCNE definition 2013.. Int. J. Clin. Pharm..

[120] Flood C., Bowers L., Parkin D. (2008). Estimating the costs of conflict and containment on adult acute inpatient psychiatric wards.. Nurs. Econ..

[121] Zhang J., Harvey C., Andrew C. (2011). Factors associated with length of stay and the risk of readmission in an acute psychiatric inpatient facility: A retrospective study.. Aust. N. Z. J. Psychiatry.

[122] Zeller S., Citrome L. (2016). Managing agitation associated with schizophrenia and bipolar disorder in the emergency setting.. West. J. Emerg. Med..

